# Experimental Assessment of Color Deconvolution and Color Normalization for Automated Classification of Histology Images Stained with Hematoxylin and Eosin

**DOI:** 10.3390/cancers12113337

**Published:** 2020-11-11

**Authors:** Francesco Bianconi, Jakob N. Kather, Constantino Carlos Reyes-Aldasoro

**Affiliations:** 1 Department of Engineering, Università degli Studi di Perugia, Via Goffredo Duranti 93, 06125 Perugia, Italy; 2 giCentre, School of Mathematics, Computer Science & Engineering, City, University of London, Northampton Square, London EC1V 0HB, UK; reyes@city.ac.uk; 3 Department of Medical Oncology and Internal Medicine VI, National Center for Tumor Diseases (NCT), University Hospital Heidelberg, Im Neuenheimer Feld 400, 69120 Heidelberg, Germany; jakob.kather@nct-heidelberg.de

**Keywords:** histology images, H&E staining, color, texture

## Abstract

**Simple Summary:**

The appearance of histology images stained with H&E can vary a lot as a consequence of changes in the reagents, staining conditions, preparation procedure and acquisition system. In this work we investigated whether color preprocessing—specifically color deconvolution and color normalization—could be used to correct such variability and improve the performance of automated classification procedures. Experimenting on 11 datasets, 13 image descriptors and eight color pre-processing methods we found that doing no color preprocessing was the best option in most cases.

**Abstract:**

Histological evaluation plays a major role in cancer diagnosis and treatment. The appearance of H&E-stained images can vary significantly as a consequence of differences in several factors, such as reagents, staining conditions, preparation procedure and image acquisition system. Such potential sources of noise can all have negative effects on computer-assisted classification. To minimize such artefacts and their potentially negative effects several color pre-processing methods have been proposed in the literature—for instance, color augmentation, color constancy, color deconvolution and color transfer. Still, little work has been done to investigate the efficacy of these methods on a quantitative basis. In this paper, we evaluated the effects of color constancy, deconvolution and transfer on automated classification of H&E-stained images representing different types of cancers—specifically breast, prostate, colorectal cancer and malignant lymphoma. Our results indicate that in most cases color pre-processing does not improve the classification accuracy, especially when coupled with color-based image descriptors. Some pre-processing methods, however, can be beneficial when used with some texture-based methods like Gabor filters and Local Binary Patterns.

## 1. Introduction

Digital pathology plays a fundamental role in cancer diagnosis, treatment and follow-up [1,2,3,4,5,6,7,8,9]. This consists of a range of activities such as the acquisition, storage, sharing, analysis and interpretation of histological images [10]. In this domain, computer-assisted classification of tissue samples has attracted considerable research interest in recent years as a means for assisting pathologists in several tasks, for instance, the classification of specimens into normal or abnormal [11,12,13,14], the grading of neoplastic tissue [15,16,17,18], the estimation of tumor proliferation [19] and the identification of tissue substructures such as epithelium, stroma, lymphocytes, necrosis, etc. [20,21]. With the growing popularity of whole-slide scanners, and consequently, the increasing availability of digital images, digital pathology has the potential not only to reduce the workload by automating several repetitive tasks, but also to increase the reproducibility of human-based evaluations.

Among the problems that so far have limited the adoption of digital pathology on a wide scale are differences in the protocols, materials and procedures for image acquisition and the little availability of large datasets of annotated images [22]. Such variations in protocols, materials and procedures can result in unlike visual appearance of the pathology slides, which can have the undesired effect of reducing the accuracy, sensibility and specificity of automated, machine-based approaches [23,24]. The problems related to stain normalization have generated considerable research interest in the last few years and several methods have been proposed in the literature [22,23,25,26,27,28,29,30,31]. However, few studies investigated the subject on a quantitative basis, and some reported divergent results. Furthermore, many such studies were based on a limited number of data sets—as few as one in some cases—which makes it difficult to draw general conclusions. Consequently, the effects of pre-processing methods on automated classification of H&E-stained images are not entirely clear yet. In [32,33] the authors reported improved accuracy for patch-based classification based on Convolutional Neural Networks (CNN), whereas [34] showed that color features lost distinctiveness when color normalization was applied. More recently, Hameed et al. [35] also reported that their classification performance deteriorated upon using color-normalized images. Furthermore, the combined effects color pre-processing/image descriptors (e.g., color descriptor, texture descriptors and/or convolutional network) have been addressed only in [34,36,37].

This work presents a quantitative evaluation of color deconvolution and color normalization on automated (patch-based) classification of histology images stained with hematoxylin and eosin from breast, prostate, colorectal cancer and malignant lymphoma. The present study extends the preliminary results presented in [38] and the main contribution is to provide a set of guidelines to select the appropriate combinations color pre-processing/image descriptor for histopathological image analysis. We found that in most cases color pre-processing did not improve classification accuracy, especially when coupled with color-based image descriptors convolutional networks. Some pre-processing methods, however, provided some slight gain when used with texture-based methods like Gabor filters and Local Binary Patterns. On the whole the best combinations involved the use of pre-trained networks (ResNet50/101) or color histograms as image descriptors and no color pre-processing at all.

## 2. Materials

**Figure 1:**
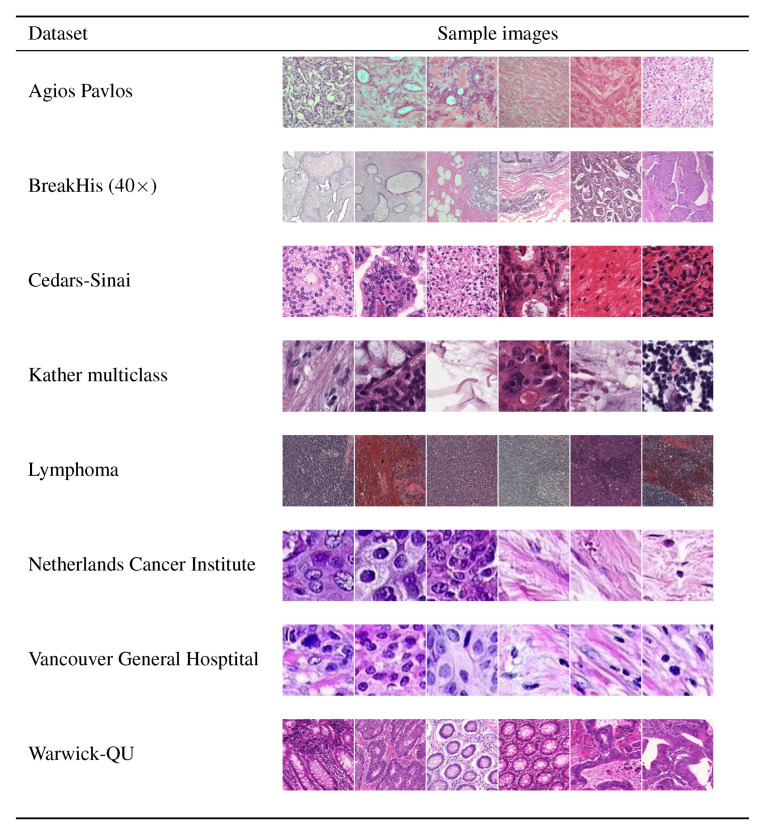
Six representative sample images from the datasets used in the experiments. It should be noticed the diverse gamut of colors as well as the different magnifications, density and cell density of the datasets. Cedars-Sinai by courtesy of Cedars-Sinai Medical Center (©2020 Cedars-Sinai Medical Center. All rights reserved).

We considered nine datasets of H&E-stained histological images representing different types of neoplastic diseases as detailed below. Samples images of each dataset are illustrated in Figure 1.

### 2.1. Agios Pavlos (AP)

Histological images from breast carcinoma collected within the ‘Agios Pavlos’ Department of Pathology at the General Hospital of Thessaloniki (Thessaloniki, Greece). The dataset includes 300 images (magnification 40×, dimension 1280 px × 960 px) of invasive ductal carcinoma (grades I, II and III) from 21 patients.

### 2.2. BreakHis (BH)

Histological samples of breast carcinoma collected at the Pathological Anatomy and Cytopathology Laboratory (P&D Lab, Paraná, Brazil) [39]. This collection features 7909 microscopy images of breast tumor tissue from eight different histological sub-types. The tissue samples were collected from 82 patients under four magnifying factors: 40×, 100×, 200× and 400×, of which the first was the one used in this study. The dimension of the images is 700 px × 460 px.

### 2.3. Cedars-Sinai (CS)

Histological images from patients with prostate cancer collected at the Cedars-Sinai Medical Center (Los Angeles, CA, USA) [40]. The data set features 625 images of dimension 1201 px × 1201 px each containing manually annotated regions of either benign tissue, stroma and/or malignant tissue (Gleason grade from III to V). The spatial resolution is ≈0.5 μm/px. From this set we randomly extracted 256 px × 256 px tiles representing clearly identifiable areas of each grade (100 tiles for each class).

### 2.4. HICL

Histological samples from 109 subjects with breast ductal carcinomas who received a biopsy at the Department of Pathology, University Hospital of Patras, Rio, Greece, between 2000 and 2007 [41]. The dataset comes with a manually defined, ground truth subdivision into grade I (n=63), II (n=83) and III (n=80). The images were acquired with 40× magnification factor and the final dimension is 1728 px × 1296 px.

### 2.5. Kather Multiclass (KM)

A dataset of histological images of colorectal cancer collected at the University Medical Center Mannheim, Heidelberg University (Heidelberg, Germany) [21,42]. The data set is composed of 5000 tissue samples (tiles) from 10 patients representing eight different tissue sub-types (see Figure 1 for details). Each tile has a dimension of 150 px × 150 px and spatial resolution of ≈0.5 μm/px. The images were acquired under 20× magnification using an Aperio ScanScope (Aperio/Leica biosystems).

### 2.6. Lymphoma

Histological images of malignant lymphoma from different institutions [43,44]. This data set is part of the Benchmark Suite for Biological Image Analysis (IICBU 2008). It includes a total of 374 images organized in three classes: chronic lymphocytic leukemia (*n* = 113), follicular lymphoma (*n* = 139) and mantle cell lymphoma (*n* = 122). The dimension of the images is 1388 px × 1040 px. Since the samples come from different centers there is a large amount of staining variation.

### 2.7. Netherlands Cancer Institute (NKI)

Breast cancer histology images from a population of 248 patients. The images were collected at the Netherlands Cancer Institute (Amsterdam, Netherlands) [45,46]. From the predefined segmentation into epithelium and stroma which comes with the dataset we respectively extracted 1106 and 189 tile images of each class (dimension 100 px × 100 px).

### 2.8. Vancouver General Hospital (VGH)

This dataset has the same structure as the one described in Section 2.7, but in this case the study population comprises 328 subjects enrolled at Vancouver General Hospital (Vancouver, BC, Canada) [45,46]. With the same procedure and settings described in Section 2.7 we extracted 226 image samples of epithelium and 47 of stroma.

### 2.9. Warwick-QU (WR)

This dataset includes a total of 165 images representing colorectal tissue and is organized in two classes: benign (*n* = 74) and malignant tissue (*n* = 91). The samples were collected at the University Hospitals Coventry and Warwickshire (Coventry and Rugby, United Kingdom) [47,48]. The images were acquired at 20× magnification factor and spatial resolution of ≈0.62 μm/px; the dimension is variable. The data set was part of the Gland Segmentation Challenge Contest (GlaS) at MICCAI 2015 (Munich, Germany, 5–9 October 2015) [49].

### 2.10. Combined Datasets (AP+HICL, NKI+VGH)

One important factor that can affect the colors of histological images are the specific conditions of the acquisition laboratory. To assess the effects of inter-laboratory variability, we generated two additional datasets by merging Agios Pavlos and HICL (AG+HICL) and NKI and VGH (NKI+VGH). These datasets were selected as they consider the same disease type and grades, and have compatible magnification factor and image resolution.

It should be noted that the images considered in this work are considerably smaller than those provided by whole-slide scanners [50,51]. Images from whole-slide scanners can span tens or hundreds of thousands of pixels, and these are typically cropped into smaller tiles and thus very large number of images can be used for studies. For reproducibility, we used the nine publicly available datasets described above.

## 3. Methods

### 3.1. Color Pre-Processing

**Figure 2:**
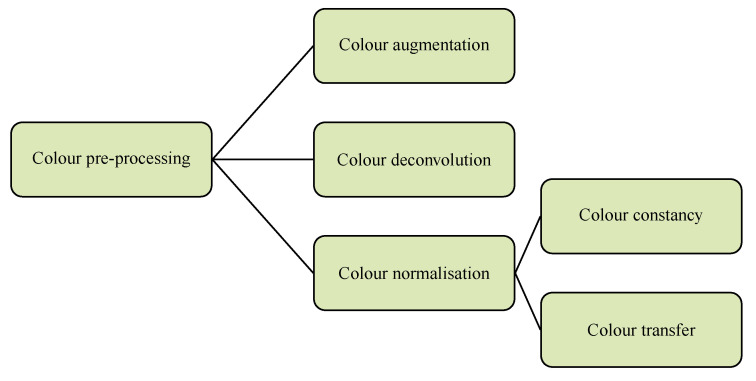
Color pre-processing for histological images: a taxonomy.

It is convenient to classify color pre-processing methods for histological images into three categories: *color augmentation*, *color deconvolution* and *color normalization* (Figure 2).

#### 3.1.1. Color Augmentation

Color augmentation is a type of data augmentation whereby new images are generated by applying some kind of perturbation to the colour distribution of the original ones [23,36]. Color augmentation was not considered in this study as it is intrinsically different from color deconvolution and color normalization, which were considered. The main difference is the input/output relationship: in both color deconvolution and color normalization, the relationship is one-to-one, while in color augmentation it is one-to-many. The number of output images returned by color augmentation is a parameter to set and depends on the method chosen. Testing color augmentation would therefore require a rather different set-up than the one used for color deconvolution and color augmentation.

#### 3.1.2. Color Deconvolution

**Figure 3:**
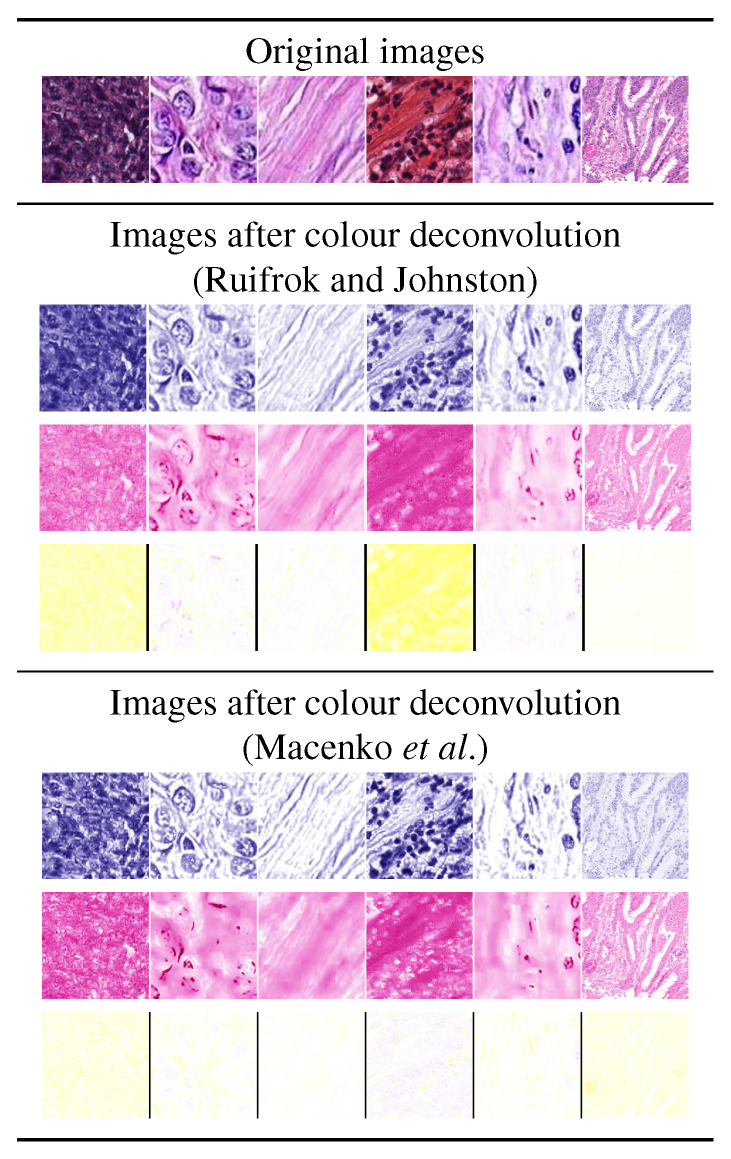
Effects of colour deconvolution through Ruifrok and Johnston’s [26], and Macenko et al.’s method [25]. The top row shows the original images, then each box below reports the deconvolved haematoxylin channel (first row), the deconvolved eosin channel (second row) and the background channel (third row). The haematoxylin, eosin and background channels are rendered in pseudo-colors.

Color deconvolution consists of decomposing the input images into separate channels, each representing the concentration of each stain used [52]. In H&E-stained images that means separating the original images into haematoxylin, eosin and background. Please note that in some cases colour deconvolution is just one step towards colour normalization [22]. In this work we considered Ruifrok and Johnston’s method [26] (‘decoRJ’ in the remainder) and Macenko’s et al. [25] (‘decoMC’ in the remainder)—both through the implementation provided in [53]. Figure 3 shows the effects of these methods on a set of sample images.

#### 3.1.3. Colour Normalization

Color normalization can be further classified into *color constancy* and *color transfer*. The first derives from color constancy in vision theory, the objective of which is to assign a constant color to the same objects when acquired under different illumination conditions [54,55]. This extends seamlessly to histological images, even if, in this case, changes in color can be due both to variable illumination and, to a greater extent, to differences in tissue preparation and staining. The second, color transfer, modifies the color distribution of the input image to make it match that of a *target* image [56]. Below we describe the color constancy and color transfer methods considered in the experiments.

The colour constancy methods investigated in this work were: (1) chromaticity representation (‘chroma’ in the remainder), (2) grey-world normalisation (‘gw’) and (3) histogram equalization (‘heq’) [57,58]. The first simply divides the *R*, *G* and *B* values of each pixel of the input image by their sum R+G+B. The second works on the assumption that the average color in a scene is grey, and that deviations of the average color from grey are due to the light source. The input image is corrected accordingly. The third modifies the marginal distribution (histogram) of each color channel by making it approximate a uniform one. The implementation was based on the Color Constancy toolbox [59] (for chroma and gw) and Matlab’s histeq() function histogram equalisation.

**Figure 4:**
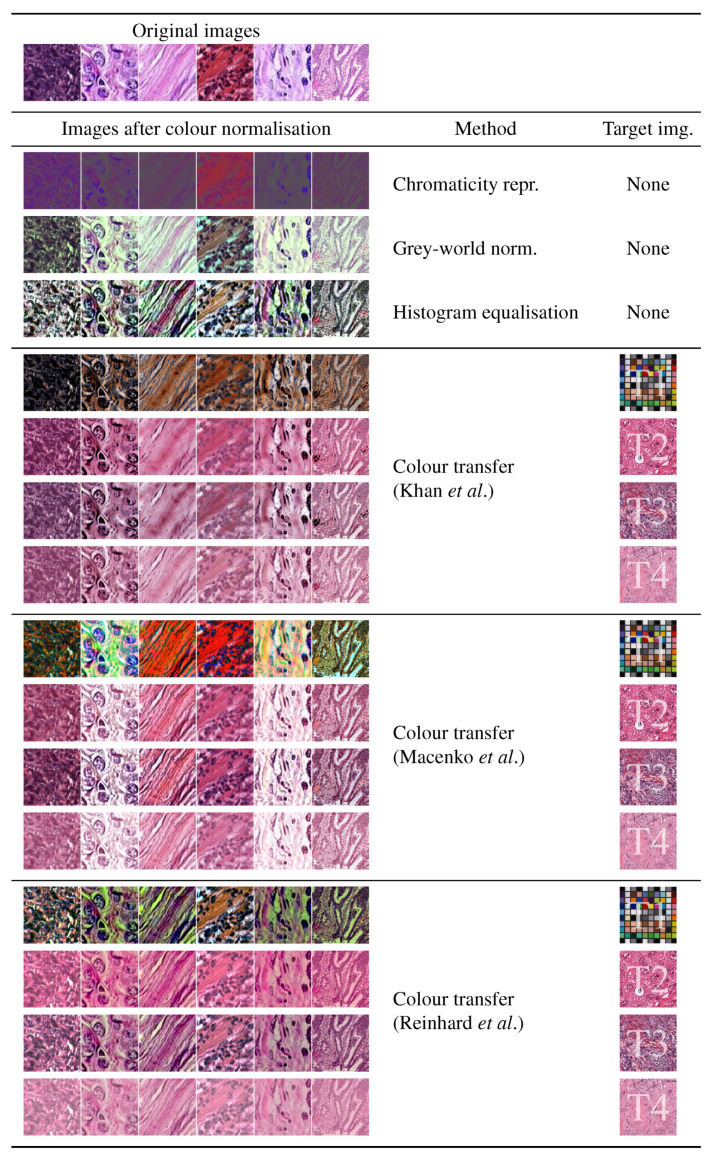
Illustration of the effects of color constancy and color transfer on a series of representative images with four different target images. Three targets are histological images, and one is a color checker mask used to investigate the impact caused by an image with a large and distant color variation.

For color transfer we considered the methodologies of Khan et al. [22], Macenko et al. [25] and Reinhard et al. [56], each with four different target images denoted as T1–T4 in the remainder (see also Figure 4). Three of these images (T2–T4) are histology images, and one (T1) is not. For the latter we selected a color calibration mask (colour checker), which is an image with a large variation of colors not related to histology. The rationale was to investigate how widely the colors of the original image could vary if those of the target image were markedly different. For the implementation we used the functions available in Warwick’s Stain Normalization Toolbox [53]. Figure 4 illustrates the effects of each color normalisation methods on a set of sample images.

### 3.2. Image Descriptors

**Figure 5:**
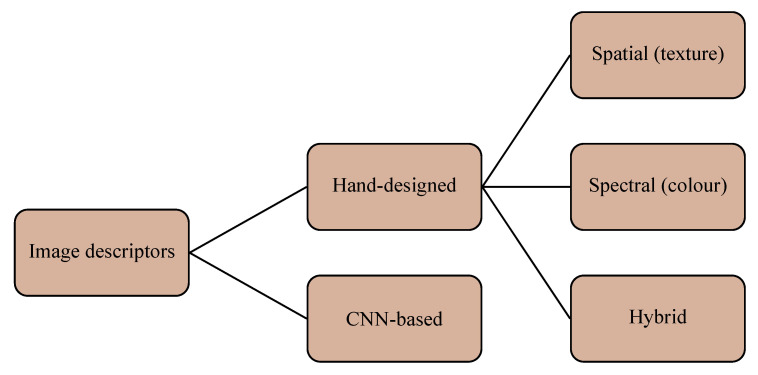
Taxonomy of the image descriptors used in this study.

The image descriptors that can be used for histological image analysis fall into two main categories: the traditional, ‘hand-designed’ methods on the one hand and the convolutional networks (CNN) on the other [60]. The first group can be further subdivided into spatial (texture), spectral (color) and hybrid methods [61] (Figure 5). For this study we considered eight ‘hand-designed’ descriptors and five pre-trained convolutional networks as detailed below.

#### 3.2.1. Hand-Designed Methods (Spectral)

##### Three-Dimensional Color Histogram (*FullHist*)

The three-dimensional probability distribution in the color space as described in [62]. We used ten bins for each color channel giving a total of $10^{3}=1000$ features.

##### One-Dimensional Marginal Color Histograms (*MargHists*)

The concatenation of the three one-dimensional probability distributions of the intensity level in each color channel [63]. We used 256 bins for each color channel giving a total of 256×3=768 features.

#### 3.2.2. Hand-Designed Methods (Spatial)

##### Grey-Level Co-Occurrence Matrices (*GLCM*)

Texture features from 12 co-occurrence matrices computed using three distances (1 px, 2 px and 3 px) and four orientations (0, 45, 90 and 135). From each matrix we extracted five statistical parameters: contrast, correlation, energy, entropy and homogeneity [64] for a total of 12×5=60 features. We finally applied Discrete Fourier Transform (DFT) normalization to obtain rotationally invariant features [65].

##### Gabor Filters (*Gabor*)

Texture features from a bank of 24 Gabor filters with four frequencies and six orientations. From the absolute value of each Gabor-transformed image we computed the mean and standard deviation giving a total 2×4×6=48 features. Again, rotationally invariant features were finally obtained via DFT normalization [66]

##### Local Binary Patterns (*LBP*)

Histograms of rotation-invariant (‘ri’) Local Binary Patterns [67] computed using non-interpolated circular neighborhoods of eight-pixels each and resolution 1 px, 2 px and 3 px (see also [68] for details). This configuration produces 36 features for each resolution, therefore a total of 36×3=108 features.

#### 3.2.3. Hand-Designed Methods (Hybrid)

From the grey-scale texture descriptors described in Section 3.2.2 we derived marginal color versions by applying the grey-scale methods to each color channel separately and concatenating the resulting feature vectors. Consequently, the marginal color versions of Gabor, GLCM and LBP (which we indicate as ‘MargGabor’, ‘MargGLCM’ and ‘MargLBP’ henceforth) have feature vectors that are three times longer than those of the grey-scale counterparts.

#### 3.2.4. Pre-Trained Convolutional Networks

We used five pre-trained convolutional networks ‘off-the-shelf’—i.e., without any further re-training or fine-tuning (see also [60,69] for details on this approach). For all the models the imaging features were the $L_{2}$-normalized output of the layer indicated in Table 1. The number of features generated by each configuration is also reported in the table.

### 3.3. Further Pre-Processing Steps

Convolutional networks have input fields of fixed shape and size, which requires the input images to be resized accordingly. To this end we cropped non-square images to a maximal centered square, then linearly resized the resulting crop to the networks’ input field. Since all the networks considered here feature a square input field, the first step was required to avoid distortion. For fair play the crop was applied in any case, even though the hand-designed descriptors can cope with input images of any shape and size. Linear resize after crop was used with the networks only.

## 4. Experiments

To test the effectiveness of each combination of color pre-processing/image descriptor (Section 3) we conducted a series of supervised image classification experiments, each of them using the data sets previously described in Section 2. We estimated the accuracy through split-sample validation with stratified sampling; that is, for each data set analyzed, we considered a fraction (*f*) of the samples of each class (i.e., the train set) to construct the classifier, and then, the remaining samples (i.e., the test set) were used to calculate the accuracy. Thus, the accuracy was the percentage of samples of the test set classified as correct. To obtain a stable estimation, we repeated the random subdivision of the train and test sets hundred times and the results were averaged. We repeated the experiments using f=1/4 and f=1/8. The classification was based on the rule of nearest-neighbor with the $L_{1}$ (‘cityblock’) distance.

The experiments were implemented using Matlab^®^ (The Mathworks^TM^, Natick, USA) and carried out on a laptop PC equipped with Intel^®^ core^TM^ i5-3230M CPU@ 2.60GHz, 8 GB RAM and Windows 7 Professional 64-bit. Feature extraction was based on the freely available Color And Texture Analysis Toolbox for Matlab (CATAcOMB) [73] for the hand-designed descriptors, on MatConvNet [74] for the ResNet and VGG models and on Matlab’s dedicated plug-in for InceptionV3.

## 5. Results and Discussion

### 5.1. Accuracy

The results for the best and second-best combinations of image descriptor and color pre-processing method for each data set are presented in Table 2. It can be observed that out of the 11 best combinations, 7 cases corresponded to the pre-trained ResNet50 and ResNet101, three cases to the joint and marginal color histograms and one to co-occurrence matrices. When considering the best and second-best cases, these corresponded to the pre-trained ResNet50 and ResNet101 in 12 cases out of 22. Regarding color pre-processing, doing nothing provided the best or second-best option in ten cases out of 22, followed by deconvolution (five) and chromaticity representation (three).

**Figure 6:**
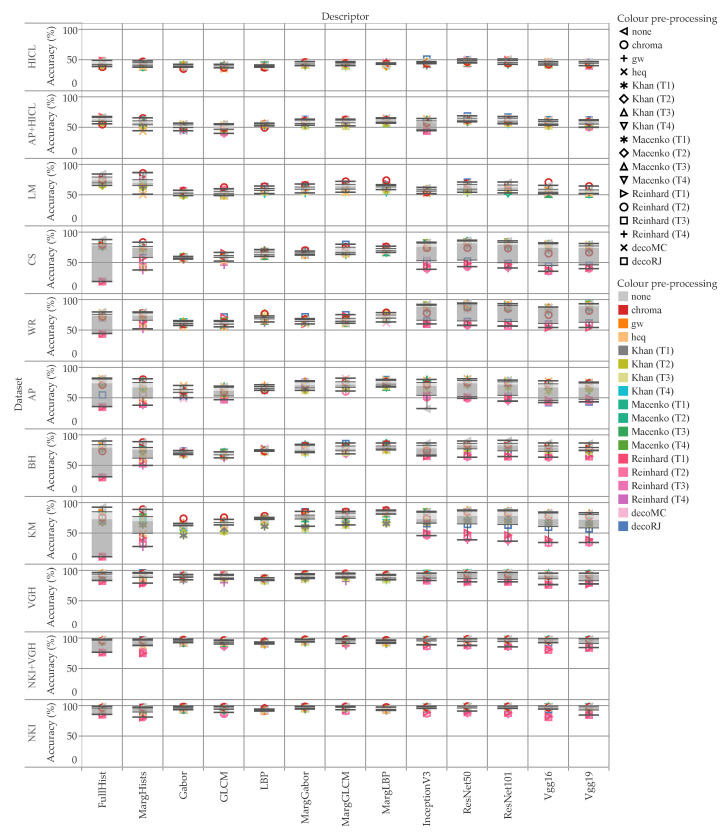
Accuracy by data set and descriptor; color indicates the pre-processing method. The values reported are for f=4. This chart shows interesting things. First, texture-based image descriptors (e.g., LBP) are much more insensitive to color pre-processing than the other methods (e.g., FullHist). This is important when analyzing the reproducibility of the methodologies. Second, the cases where there was large variation seemed to have results on the extremes (i.e., KM / FullHist) and not a uniform distribution. Third, and perhaps the most important, the accuracy obtained in different datasets is considerably different. Compare for instance NKI which is very close to 100% with LM where most cases are around 50%. This highlights the importance of testing on more than one dataset, as the choice of dataset can result in higher or lower values of accuracy.

Figure 6 shows the accuracy for each descriptor and data set, while color indicates the pre-processing methodology. As can be observed, the performance of the color-based descriptors (i.e.: color histograms and pre-trained networks) varied significantly depending on the pre-processing method used. By contrast, the texture-based descriptors were markedly more resilient, as one would reasonably expect. Also, it should be noted that the marginal versions of the texture descriptors (MargGabor, MargGLCM and MargLBP) outperformed their grey-scale counterparts (Gabor, GLCM and LBP).

**Figure 7:**
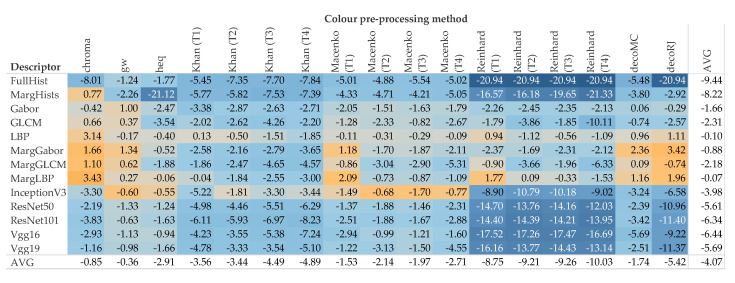
Difference to the baseline by image descriptor and pre-processing method. The values are averaged over the eight data set and filtered on f=4. Baseline is the condition where no color pre-processing is applied.

**Figure 8:**
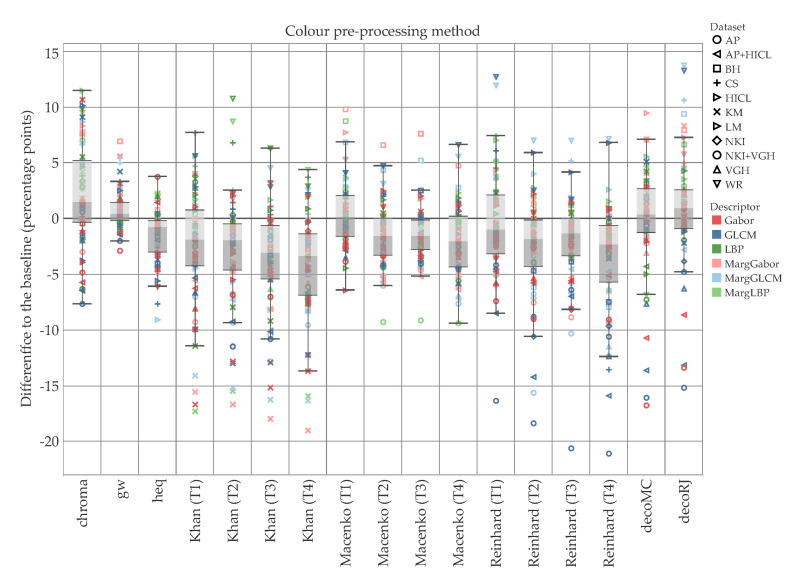
Difference to the baseline for each of the color pre-processing methodologies (texture and hybrid hand-designed descriptors). Color shows details about descriptor, shape about data set. The data are filtered on f=4. The zero line represents the condition where no color pre-processing was applied. The use of color pre-processing caused loss of accuracy in the majority of the cases: the median of 12 of the 17 methodologies was below the baseline, and the upper quartile of 11 of the 17 was close or below the baseline. It should be noted that for both Macenko and Reinhard, the best results were recorded when T1—i.e., the non-histological target—was used. Ruifrok and Johnston’s (decoRJ) was among the highest results, together with the relatively simpler chroma and gw.

**Figure 9:**
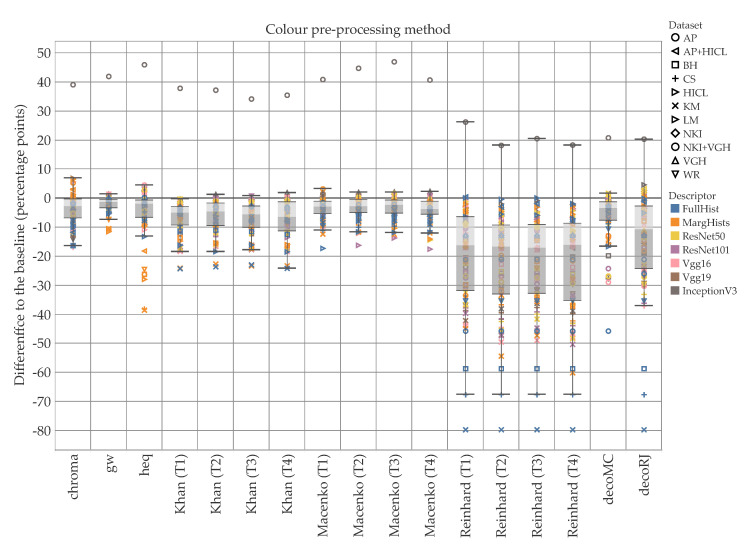
Difference to the baseline for each color pre-processing method (color hand-designed descriptors and convolutional networks). Color shows details about descriptor, shape about data set. The data are filtered on f=4. The zero line represents the condition where no color pre-processing was applied. It should be noticed the considerable decrease of accuracy of Reinhard’s methodology, irrespective of the target image. This is due to the reliance of the methodology on color. On the other hand, results for T1 were slightly higher than T2-T4 for both Reinhard and Macencko. This is surprising as T1 is not histological and the colors are considerably different from the images to normalize. Note the presence of outliers both above and below the zero line, respectively InceptionV3/AP and FullHist/KM.

Figure 7 reports the difference to the baseline (i.e., no color pre-processing) divided by descriptor and color pre-processing methodology. These values are averaged over all the data sets. The box plots of Figure 8 and Figure 9 break down the difference by color pre-processing method, while color and shape of the markers respectively show details about the descriptor and data set. On the whole, color pre-processing caused a loss of accuracy in most cases. This was particularly true when pure color descriptors and convolutional networks were involved (Figure 9); moreover, we can see that in some cases the decrease in accuracy was very sharp. Those methodologies which rely heavily on color responded negatively to color pre-processing, which is in line with the results reported in [34]. The results also show that the outcome of color transfer methodologies (Khan’s, Macenko’s and Reinhard’s) was pretty much independent on the target image used, regardless this being a histology image (T2–T4) or not (T1). In fact, it is quite surprising that on average T1 performed slightly better than the others (Figure 7). We believe this is an important finding, because it suggests that despite the color-transformed images obtained using T2–T4 as target images ‘look better’ than those obtained using T1, this does not translate into a better performance of the automatic classification. A comparison among the three methods show that Khan’s and Macenko’s had a similar performance, whereas that of Reinhard’s was markedly worse. Regarding color deconvolution, we observe (Figure 7) that on average this was generally beneficial only when coupled with texture descriptors, but not in the other cases (i.e., color descriptors and pre-trained CNN).

The methods based on texture proved fairly resilient to color pre-processing (Figure 6), as it would reasonably expected. In these cases, there was even a noticeable improvement of the accuracy in some combinations of the descriptor and the pre-processing methodology. Specifically, the marginal color texture descriptors (i.e., MargGabor, MargGLCM and MargLBP) seemed to provide a positive response both to ‘chroma’ normalization and color deconvolution. The latter results looked particularly interesting, i.e., it suggests that the texture features can provide complementary information when applied to each of the channels separately, i.e., haematoxylin, eosin and background.

To reduce potential sources of bias related to the samples distribution in the training and test sets, we repeated the classification experiments using a lower train ration (f=1/8). The complete results show that no significant difference was observed with the trend with f=1/4.

### 5.2. Computational Demand

**Figure 10:**
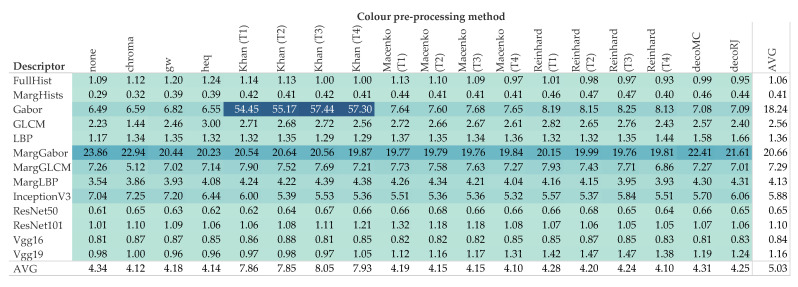
Feature extraction time (sec/image). The values were recorded on the HICL dataset. Please note that for efficiency reasons, the color-preprocessed images were cached after the first calculation, which was carried out during the extraction of ‘Gabor’ features. Therefore, the figures in the ‘Gabor’ row include both the color pre-processing time and the feature extraction time.

Figure 10 illustrates the average feature extraction time by descriptor and color pre-processing methods. On the whole the results indicate that there was some additional overhead, as one would reasonably expect. This was more noticeable for the color transfer methods—particularly Khan’s—than for the color constancy ones, which is consistent with the higher complexity of the first group compared with the second. Surprisingly, there was a gain in speed in some cases, as for instance with the combinations chroma normalization/GLCM and MargGLCM. A possible explanation is that by definition, chroma normalization projects the color distribution onto a plane, therefore effectively reducing the dimensionality of the color space from three to two. As for the image descriptors, it can be seen that MargHists was the quickest method, followed by FullHist, LBP and the ResNet and VGG pre-trained models. The other texture descriptors were significantly slower, as was InceptionV3.

## 6. Conclusions

Digital pathology is a rapidly developing discipline with important implications, for instance, the management of those patients who present neoplastic disorders. Potential applications include disease classification, identification of blood vessels, mitosis detection and tissue segmentation. Crucial to all them is the classification of tissue areas into homogeneous and clinically significant regions. As a result of immuno-staining, color plays a significant role in this process, for it enables the differential visualization of tissue micro-structures such as nuclei, ribosome and cytoplasm. However, variations in tissue preparation, reagents, image acquisition settings and other factors can easily lead to significant differences between whole-slide images. To circumvent these problems several pre-processing methodologies have been investigated. Although such procedures can produce appealing results on a qualitative basis, their effects on automatic patch-based classification of histological slides are not clear.

In this work we found that color pre-processing resulted in a noticeable reduction of the accuracy in most cases, especially when coupled with image descriptors that rely heavily on the color of the image. This agrees with the results presented in [34], but differ from those appeared in [33]. In [35,36] the authors achieved the top performance without the use of color normalization, which is again consistent with the results found here. Our findings also conform with those reported by Cusano et al. [55] for the recognition of color textures under variable lighting conditions—a problem conceptually equivalent to the one investigated here. Interestingly, some pre-processing methods (i.e., chroma and decoRJ) provided positive effects when joined with certain texture descriptors, i.e., MargGabor, MargGLCM and MargLBP. We consider that this is a novel finding that could pave the way to new investigations in future studies.

We speculate that the most interesting new investigations would be those that follow the impact of color pre-processing and pass the classification stage towards the correlation with clinical outcome. Currently, there are several reports that correlate clinical outcome with bio-markers derived from histological images [50,51,75,76,77,78], and while these studies provide promising results, it would be interesting to test if these could be affected by color pre-processing.

In conclusion, the results suggest that the application of color pre-processing methodologies for patch-based classification of H&E-stained images should be considered with care. Although our results show some dependence on the dataset used, on the whole our findings indicate that in the absence of enough data for domain-specific tuning, (1) doing nothing (no color pre-processing) is likely to be a good option in most cases (*primum non nocere*) and (2) pre-trained CNN from the ResNet family are the descriptor of choice. Otherwise, if there are enough data enough to carry out some domain-specific tuning, we recommend the color pre-processing method(s) be always evaluated along with the image descriptor(s) used.

## Figures and Tables

**Table 1:** Round-up table of the pre-trained convolutional models considered in the study.

Model	Ref.	Layer (Name/No.)	No. of Features
InceptionV3	[70]	313	2048
ResNet50	[71]	‘pool5’	2048
ResNet101	[71]	‘pool5’	2048
Vgg16	[72]	‘FC-4096’	4096
Vgg19	[72]	‘FC-4096’	4096

**Table 2:** Best (rank = 1) and second-best (rank = 2) combinations color pre-processing/image descriptor by dataset. Figures indicate accuracy, also reflected in the ball size and color (blue = low, brown = high). Values are filtered on f=1/4.

Dataset	Rank	Accuracy (%)	Descriptor	Pre-Processing
AP	1	81.79	MargGLCM	decoMC
	2	81.70	FullHist	heq
AP+HICL	1	68.97	ResNet50	decoRJ
	2	67.61	FullHist	Reinhard (T1)
BH	1	90.67	ResNet101	none
	2	90.07	ResNet50	none
CS	1	87.59	FullHist	none
	2	86.39	ResNet50	none
HICL	1	51.58	ResNet101	decoMC
	2	51.51	InceptionV3	decoRJ
KM	1	92.18	FullHist	none
	2	89.03	MargHists	chroma
Lymphoma	1	85.98	MargHists	chroma
	2	84.53	FullHist	none
NKI	1	98.87	ResNet50	none
	2	98.86	ResNet50	chroma
NKI+VGH	1	98.39	ResNet50	none
	2	98.33	ResNet101	gw
VGH	1	96.10	ResNet101	none
	2	96.00	MargHists	decoRJ
WR	1	94.37	ResNet50	none
	2	94.11	ResNet50	Khan (CC140)

## Abbreviations

The following abbreviations are used in this manuscript:

CNN	Convolutional Neural Network(s)
GLCM	Grey-level Co-occurrence Matrices
H&E	Hematoxilyn & Eosin
LBP	Local Binary Patterns
TCGA	The Cancer Genome Atlas
TMA	Tissue Micro-array(s)
